# Case Report: The risks associated with chronic theophylline therapy and measures designed to improve monitoring and management

**DOI:** 10.1186/s40360-016-0050-4

**Published:** 2016-03-05

**Authors:** Michael E. Hopkins, Robert V. MacKenzie-Ross

**Affiliations:** Foundation Year 1 Doctor. Royal United Hospital NHS Foundation Trust, Combe Park, Bath, BA1 3NG UK; Consultant Respiratory Physician. Royal United Hospital NHS Foundation Trust, Combe Park, Bath, BA1 3NG UK

**Keywords:** Theophylline, Toxicology, Pharmacokinetics, Drug Interactions, Drug adverse effects

## Abstract

**Background:**

Symptoms of theophylline toxicity and factors that augment the risk of developing it are well documented in the literature. However these appear to be poorly considered in clinical practice. This case underlines the challenges in recognising and managing theophylline toxicity; moreover the requirement for improved application of knowledge of its pharmacokinetics to our practice.

**Case Presentation:**

In this case we observe how theophylline toxicity can be overlooked due to the presence of non-specific symptoms and lack of a structured system to mitigate error in detection, in both hospital medicine and general practice. Here, the initial theophylline concentration measurement was documented as 59.3 mg/l in a patient taking the medication long-term, with the previous concentration being recorded one year prior. Management consisted of suspension of theophylline along with best supportive care, however in the process other conditions were exacerbated and the patient ultimately died in hospital. Congestive cardiac failure, congestive liver disease and polypharmacy were factors isolated from this case that expedited the patients’ development of theophylline toxicity. This was perpetuated by delay in diagnosis due to presentation with generalised symptoms including tachycardia, vomiting and neurological symptoms.

**Conclusions:**

Findings from this case necessitate a requirement for more stringent monitoring of theophylline when taken chronically in those who demonstrate risk factors for toxicity. This would aim to prevent accumulation of the drug, toxicity onset and subsequent acute presentation to hospital. Intervention, through charcoal haemoperfusion may provide a means of enhanced recovery to reduce sequelae of toxicity.

## Background

Theophylline is a member of the methylxanthine class of medications and is typically used in the management of patients with airway disorders such as Chronic Obstructive Pulmonary Disease (COPD) and asthma. This is due to its relaxing effect on smooth muscle within the bronchi and pulmonary vasculature, as well as stimulating central respiratory drive [1]. Theophylline is used in the management of reversible airways obstruction or exacerbations of COPD [2], it is typically an adjunct to other, more commonly prescribed medications, such as beta-2 agonists and steroids. The dose is typically started at 225 mg BD and then increased to 450 mg BD after one week. Classically, theophylline is not prescribed as a first-line medication due to its narrow therapeutic index, the British National Formulary (BNF) suggest target plasma concentrations of 10–20 mg/l [2].

This case outlines an example of the severity of adverse effects demonstrated by theophylline; furthermore we explore the pitfalls in recognising theophylline toxicity, particularly when taking the medication chronically. Many of the typical symptoms of theophylline toxicity are non-specific, which further perpetuates the challenges of recognition in a long-term user. These include nausea, vomiting, fatigue, tachycardia and central nervous system (CNS) effects such as dizziness and seizures [3]. When given in the acute setting, more precautions are taken to monitor serum concentrations. However it appears we are less stringent on monitoring when the medication is taken chronically, as exemplified in this case. The BNF suggests monitoring every 6–12 months for those patients on a stable dose [4]. However significant individual variations in hepatic metabolism of the drug also contribute to a difficulty in maintaining a therapeutic concentration.

The aim of this report is to provide a case-based example to advocate more stringent theophylline concentration monitoring, particularly in patients considered high risk for toxicity. The intention is to help prevent toxicity occurring in patients taking this medication long-term.

## Case Presentation

Mrs. E was a 57-year-old lady with a 12-year history of COPD, with an FEV1 of 30 %. Furthermore, she had a past medical history of carcinoma of the breast, type 2 diabetes mellitus and left ventricular failure (LVF), which was diagnosed on an echocardiogram (ECHO) in July 2014. Despite a diagnosis of COPD she continued to smoke 20–30 cigarettes/day.

She was prescribed a number of medications to help manage her airways disease including tiotropium, seretide 500, salbutamol, prednisolone and most recently oral theophyline (450 mg twice daily), which was started in April 2013 as an adjunct to her current medications.

She was admitted in June 2015 following a fall with notable decreased sensation bilaterally in her lower limbs and a tachycardia of 133 beats per minute. Spinal cord compression was an initial concern, however this was excluded with a magnetic resonance imaging (MRI) spine. There was no change to her theophylline prescription on admission to hospital and no new medication was prescribed that would affect theophylline metabolism.

Over several days additional symptoms were noted, including worsening nausea, blurred vision, increasing fatigue (with stable blood sugars) and worsening speech with slurring of words. Development of vomiting resulted in a decision to measure her theophylline concentration and perform a CT head, as no obvious cause for her symptoms had been elicited. The CT head was reported as clear of any ischaemia, haemorrhage or space-occupying lesion.

The trough theophylline concentration was 59.3 mg/l. The last result obtained in July 2014 during an admission for influenza related infective exacerbation of COPD was reported as 20.2 mg/l, marginally above the 10-20 mg/l recommended reference range. The concentration was then not re-checked either in primary care or in hospital; it therefore became apparent this was a case of chronic theophylline toxicity.

### Management

The medical team at The National Poisons Information Service was contacted for advice on management of this patient. The rationale being abnormally high theophylline concentration, furthermore this was a case of chronic, rather than acute toxicity, for which we felt specialist input was required.

The patient was monitored for significant effects of toxicity; these include seizures (reported at trough concentrations of 50 mg/l [5]), reduced conscious level and tachyarrhythmias^.^ Although Mrs. E suffered a persistent tachycardia, her heart rate remained in sinus rhythm. Cardiac monitoring was started. She was managed conservatively by stopping the theophylline and monitoring symptoms. Due to vomiting, oral activated charcoal was excluded and although charcoal haemoperfusion was discussed, it was not accessible at the RUH and as Mrs. E was haemodynamically stable and not acidotic, it was deemed not to be essential. This point will be discussed further in the conclusion.

Mrs. E developed electrolyte imbalances not long after the vomiting started, suffering hypokalaemia (3.2 mmol/l), hypomagnaesemia (0.56 mmol/l) and hypocalcaemia (1.96 mmol/l). This was postulated to be secondary to both vomiting and theophylline accumulation. This subsequently resulted in a pseudo-obstruction, diagnosed on abdominal x-ray. A nasogastric tube was placed and she was supplemented with appropriate electrolytes via intravenous infusion. An arterial blood gas demonstrated a pH 7.398 (7.34-7.44), pO_2_ (kPa) 9.24 (11–14), pCO_2_ (kPa) 6.36 (4.7-6.0), HCO_3_^−^ (mEq/l) 21.7 (22–26), lactate (mmol/l) 1.5 (0.4-2.2). As no signs of acidosis were present and bicarbonate was at the low end of the normal range, we decided not to prescribe bicarbonate for this patient.

After stopping her theophylline and without any active intervention her serum concentration fell gradually (Table 1). By the time her theophylline concentration was within therapeutic range, her electrolytes had been corrected and her pseudo-obstruction had resolved. However, she developed signs of fluid overload, her heart rate also remained in persistent sinus tachycardia. Notably, findings of Congestive Cardiac Failure (CCF) were demonstrated on a previous echocardiogram and were substantiated by results of her computed tomography (CT) abdomen/pelvis and ultrasound sonography scan (USS) liver during her current admission; these both showed abnormal appearances of the liver consistent with cardiac dysfunction. It therefore appeared she had developed acute CCF. Ultimately, this resulted in challenging fluid balance management with serial chest x-rays demonstrating worsening bilateral effusions and renal function continuing to decline until the death of the patient.

**Table 1:** Theophylline measurements post stopping of the medication

Time after initial measurement	Theophylline Level (mg/L)
Initial Measurement	59.3
12 h	51.6
24 h	43.4
48 h	31.1
3 days	22.8
4 days	15.4
5 days	10.1

Serial theophylline readings recorded after stopping the medication. Initial reading was a trough measurement post stopping the medication. Theophylline reference range 10-20 mg/L

## Discussion

There are a number of key learning points to draw from this case, pertaining to multiple areas of clinical practice. In retrospect, it remains difficult to understand exactly to what extent theophylline toxicity contributed to the death of this patient with multiple co morbidities. However, what is clear from the literature and from this case is that toxic theophylline concentrations can result in significant morbidity with potentially fatal outcomes [6].

As a medication with a narrow therapeutic index and an inexorable correlation between plasma concentration and therapeutic/toxic effects, there should be an impetus on close monitoring of serum concentration. Theophylline is rapidly and completely absorbed after oral administration and freely distributes throughout fat-free tissues. The drug is primarily metabolised by the cytochrome P450 liver enzyme, CYP1A2, and also eliminated via the hepatic route, with a half life of 9 h [7, 8]_._ There are a number of medications and conditions that can impact on this, rendering theophylline a difficult drug to manage. One such condition is CCF with hepatic congestion, which has been demonstrated to reduce the clearance by 50 % and double the half life, thereby reducing drug elimination and increasing concentrations [5, 9, 10]. Other recognised factors which can reduce theophylline clearance include increased age and liver enzyme inhibiting medications [11, 12]. A key point that pertains to this case is that smoking is an inducer of CYP1A2. We recognise that Mrs. E continued to smoke until her final hospital admission, which theoretically would confer an increased elimination of theophylline and act as protective mechanism against the development of toxicity. To our knowledge she was receiving no medications that are inhibitors of this enzyme. However it is critical to recognise these (Table 2.) as well as identifying recent smoking cessation; as these could both significantly impact on theophylline concentrations [13].

**Table 2:** Medications that inhibit the liver enzyme CYP1A2

CYP1A2 inhibitors
Atazanavir
Cimetidine
Ciprofloxacin
Enoxacin
Ethinyl Estradiol
Fluvoxamine
Thiabendazole

List outlining some commonly prescribed medications that are recognised to inhibit CYP1A2

Indeed, we should approach treating patients with theophylline, who have underlying CCF, with increased caution. With data demonstrating that elimination time of the drug can be doubled [9, 10] with this condition, there should be a greater onus on primary care physicians, as well as hospital clinicians to monitor the serum concentration to avoid chronic intoxication. It is sub-standard practice to neglect to monitor theophylline concentrations for over a year in a patient with established CCF, particularly when they have had a previously high reading [14]. The BNF suggests that serum concentrations should be measured every 6–12 months in patients with a stable dose after 5 days [3]. However it would be reasonable to suggest that those patients who have risk factors for reduced elimination or metabolism of the drug, such as CCF or liver disease, are monitored at least every 6 months to avoid the manifestation of significant intoxication and avoid acute presentation.

When discussing with Mrs. E the reasons behind her admission, it was interesting to hear that neither she, nor her full-time carer, knew the name theophylline or its indication. In cases of polypharmacy like this, it can be challenging for patients to remember all medications, their indication and associated adverse effects. This further substantiates the argument for responsibility within healthcare professionals to provide close monitoring and support for these vulnerable patients.

Indeed there were shortcomings in patient management in this case, however it is recognising intoxication that poses one of the key challenges. This difficulty can be attributed to the generalised symptoms displayed by the patient. Neurological symptoms, fatigue, vomiting, blurred vision; these are all symptoms that could be a manifestation of multiple other disorders, especially in a patient with multiple co-morbidities. Other reports have described concomitant findings, outlining the challenges of diagnosing theophylline toxicity in hospital due to the broad array of symptoms at presentation [4]. We postulate that better education around toxicology and an increased awareness of chronic theophylline toxicity, as well as serum concentration screening for all patients who come through the emergency department taking this medication, may help improve outcomes within the hospital setting.

With regards to intervention, one study demonstrated that in cases of severe theophylline toxicity (trough concentration >48 mg/l) there was a significant reduction in morbidity and mortality to those patients who underwent charcoal haemoperfusion [15]. This increases theophylline clearance from the normal endogenous value of 50 ml/kg/h up to 157 ml/kg/h [16]. In future cases, it would be interesting to observe whether charcoal haemoperfusion would enhance recovery from theophylline toxicity and improve patient outcomes as the literature tenuously suggests [15]? At what serum concentration would you expect to see a consistently significant benefit from this therapy? Further research is required into charcoal haemoperfusion as well as incidence of theophylline toxicity before this intervention can become more widely available across hospitals.

## Conclusions

Findings from this case necessitate a requirement for more stringent monitoring of theophylline when taken chronically in those who demonstrate risk factors for toxicity. This would aim to prevent accumulation of the drug, toxicity onset and subsequent acute presentation to hospital. Intervention, through charcoal haemoperfusion may provide a means of enhanced recovery to reduce sequelae of toxicity.

## Ethics

### Consent to publish

Due to the unfortunate death of the patient in our case report, consent to publish this report was obtained from her daughter (her next of kin). A written copy of this consent form is available for review from the series editor of this journal.

Formal approval from an ethics committee has not been sought as this is a ‘Case-Report’ and therefore does no require research ethics committee approval.

### Availability of supporting data and materials

All relevant data for this case report was collected from the case notes of the patient discussed. Although confidentiality must be maintained, these case notes can be acquired from the medical records department at the Royal United Hospital, Bath.

## Abbreviations

BNF: British national formulary
CCF: congestive cardiac failure
CNS: central nervous system
COPD: chronic obstructive pulmonary disease
CT: computed tomography
ECHO: echocardiogram
LVF: left ventricular failure
MRI: magnetic resonance imaging
RUH: royal united hospital
USS: ultrasound sonography scan
